# Properties of Mechano-Transduction via Simulated Microgravity and its Effects on Intracellular Trafficking of VEGFR's

**DOI:** 10.18632/oncotarget.472

**Published:** 2012-05-06

**Authors:** Andrew Puca, Giuseppe Russo, Antonio Giordano

**Affiliations:** ^1^ Sbarro Institute for Cancer Research and Molecular Medicine, Temple University Philadelphia, PA USA; ^2^ Department of Human Pathology and Oncology, University of Siena, Nuovo Policlinico “Le Scotte”, Siena, Italy

**Keywords:** Thrombopoietin, VEGFR's, CD34+, CD45

## Abstract

This study emphasizes the dynamical properties of mechanical loading via simulated microgravity, its effect on acute myeloid leukemia proliferation and hematopoietic stem cell (HSPC) growth and its implications in the area of tissue regeneration. Data presented illustrates that mechanical transduction changes the expression of humoral factors by facilitating paracrine/autocrine signalling, therefore modulating intracellular trafficking of tyrosine kinase receptors. Understanding mechano-transduction in the context of cell and tissue morphogenesis is the major focus of this study. The effects of external physiological stresses, such as blood flow, on several cellular subtypes seem to produce very intricate cellular responses. It is well accepted that mechanical loading plays an intrinsic and extrinsic influence on cell survival. This study shows how microgravity effects hematopoietic stem cells, and human leukemic cell proliferation and expression of its receptors that control cell survival, such as the tyrosine kinase vascular endothelial growth factor receptor-1, receptor-2 and receptor-3.

## INTRODUCTION

Cells are entities in space and time. Systems Biology strives to understand their composition, structural organization as well as dynamic behavior under different conditions. Here, measures for dynamic properties such as mechanical loading, depict a hierarchy and time-dependent response in relation to these inductive properties of microgravity. Circulating cells in the blood stream have the ability to sense multiple simultaneous inputs. The integration of these signals inside the cell ultimately dictates its biological behavior. Physical forces along with biochemical signals mediated by growth factors and adhesion molecules are the fundamental regulators of tissue development. Cells may sense mechanical stresses in their local environment, such as, those due to gravity, through the balance of forces that are transmitted across trans membrane adhesion receptors that link the cytoskeleton to the extracellular matrix and to the other cells [1]. However, the mechanism by which these mechanical signals are transduced and converted to biochemical responses are not clearly understood. Recent studies suggest that cells sense mechanical stresses, including those due to gravity, through changes in the balance of forces that are transmitted across trans membrane adhesion receptors that link the cytoskeleton to the extracellular matrix and to other surrounding cells (e.g., integrin’s, cadherin’s, selectins). The mechanism by which these mechanical signals are transduced and converted into a biochemical response appears to be based, in part, on the finding that living cells use a tension-dependent form of architecture, (tensegrity) to organize and stabilize their cytoskeleton. Tensegrity allows for cellular response stress to vary depending on several factors, most importantly, pre-existing stress or tension in the cytoskeleton. This involves all three cytoskeletal filament systems as well as nuclear scaffolds. Past work on mechanotransduction has revealed that certain cells have evolved specialized crystal structures that respond directly to the force of gravity. These dense crystals are called statoliths, literally “standing stones,” or otoliths, as in the case of the sensory cells of the inner ear. When there is a movement of the human head, these dense crystals slide over the receptor cells like tiny lead weights, and it is the resulting localized distortion of the cell surface and interconnected cytoskeleton (CSK) that is somehow sensed by the cell. The statolith represents an elegant mechanism for mechanotransduction; however, it does not explain how all of the cells in the body sense gravity [2]. To understand how gravitational forces alter cell function, we must place this form of developmental control in context of what we have learned in recent years about other forms of cell regulation. Tensegrity does more than predict pattern formation. It helps explain how cells sense and respond to external mechanical signals [1, 3].

The rotating cell culture system, also known as the rotating wall vessel (RWV) system developed by NASA provides us with a novel way to see inside a cell by understanding how gravitational forces alter cell function. In this rotating wall bioreactor, the liquid media and the cells rotate with the walls of the container. This action suspends the cells in the media so that the effects of gravity-driven convection and sedimentation are significantly reduced. The two factors governing the simulated microgravity environment are, low shear stress that promotes close apposition of the cells, and randomized gravitational vectors which either affect gene expression or indirectly facilitate paracrine/autocrine intercellular signaling through diffusion of differentiative humeral factors. Through solid body rotation and viscous coupling, the RWV bioreactor subjects suspended cells to a continual state of free fall, hence, simulating microgravity [4].

The bone marrow microenvironment is extremely important in providing extrinsic signals for hematopoiesis. The bone marrow microenvironment releases soluble membrane-bound cytokines such as, interleukins and kit-ligand (KitL), to support the survival of subsets of stem cells [5]. Thrombopoietin (TPO) promotes megakaryocytopoiesis and thrombopoiesis (GM-CSF), (G-CSF) granulocyte-CSF sustains and induces the proliferation of myeloid lineages, and (EPO) erythropoietin primarily affects expansion and differentiation of the erythroid lineage, however, the identity of cytokines promoting the self-renewal of hematopoietic stem cells is not known [6]. The bone marrow extra cellular matrix (BMEC) not only acts “*in vivo*” as a gatekeeper, by controlling the trafficking and homing of hematopoietic progenitors, but also provides cellular contact and secretes cytokines that allow for the preservation for a steady state of hematopoiesis. Bone-marrow-derived stem cells play a major role in the regulation of several postnatal processes, including wound healing, organ regeneration, and tumor growth [7]. Tissue-specific stem cells reside in specific niches within the bone marrow microenvironment, where they are maintained in an undifferentiated and quiescent state [8]. These niches are crucial for regulating self-renewal and cell fate decisions, as well as providing an expendable source of stem cells for tissue vascularization and organogenesis [9, 10]. Studies have portrayed that an autocrine (endothelial-dependent) and a paracrine (endothelial-independent) loop between bone marrow endothelium and cells that reside in the bone marrow, is required in order to sufficiently provide a mode for proliferation and migration. Additionally, studies have eluded to the fact that not only VEGF signals through receptor expression VEGFR-1 (Flt-1) and VEGFR-2 (FlK-2/KDR) specifically regulate endothelial function, but are also involved in the growth regulation of subsets of tumor cells such as leukemia [11, 12].

Several general models indicate that a bone marrow cell can continually change its surface receptor expression responding to external stimuli differently at various points in the cell cycle. Changes in focal adhesions, cytoskeletal organization and gene expression are major responses of endothelial cells to shear stress [3]. There are countless significant questions to determining any patterns, specific to the genetic and epigenetic signals that regulate the fate of stem cells. Recent studies show that specific “*in vitro*” mechanical signals in specially designed bioreactors may provide important adjuncts to standard biochemical signaling pathways for promoting engineered tissue growth. Other studies which focus on a better understanding of the biochemical and biomechanical pathways involved in mechanical signal transduction by stem cells, will hopefully provide new insight for the improvement of stem-cell based therapies [2, 12, 13]. Over the past several years, there has been increasing interest in understanding stem cell biology, particularly as it relates to the control of stem cell fate. The final phenotype of the cell depends on the state of the cell and the inductive influences of the microenvironment, all functioning on competition and requiring cooperation [13].

The effects of external physiological stresses (blood flow) on hematopoietic stem precursor cells (HSPC’s) seem to produce very intricate cellular responses. Today, it is well accepted that mechanical loading plays an intrinsic influence on cell survival. For this reason, cellular acclimation to mechanical stimuli might present new insight as to how cells respond in terms of proliferation, differentiation, and migration in tissue regeneration [14, 15]. Several general models indicate that a bone marrow cell can continually change its surface receptor expression responding to external stimuli differently at various points in the cell cycle [14, 16]. There are countless significant questions as to determining any patterns, specific to the genetic and epigenetic signals, that regulate the fate of stem cells. Recent studies show that specific “*in vitro*” mechanical signals in specially designed bioreactors may provide important adjuncts to standard biochemical signaling pathways for promoting engineered tissue growth. Future studies focusing on a better understanding of the biochemical and biomechanical pathways involved in mechanical signal transduction by stem cells, will hopefully provide new insight for the improvement of stem-cell-based therapies [17, 18].

Over the past several years, there has been increasing interest in understanding stem cell biology, particularly as it relates to the control of stem cell fate. It is common opinion that stem cells hold significant promise for therapeutic applications in tissue or organ repair in the treatment of diseases such as heart disease, arthritis, and neural disorders, such as Parkinson’s disease and paralysis, as well as other pathologies related to trauma or degeneration [19]. A critical step in developing successful treatment of diseases will require a more rigorous understanding of the differentiation pathways which guide the uncommitted cell toward a specific tissue phenotype and promote the repair or regeneration of damaged or diseased tissue [2, 20-22]. This is a complicated endeavor, as the undifferentiated phenotype is governed by a myriad of factors, including the micro and macro environment of the cell, of which includes biochemical and biomechanical stimuli.

The mechanisms by which these mechanical signals are transduced and converted to biochemical responses are not clearly understood. Trafficking of stem cells is regulated through sequential interaction with chemokines and adhesion molecules. Homing of hematopoietic stem cells to the bone marrow is dependent upon a multi-step process in which tethering, mediated by E-selectin, is followed by firm adhesion mediated by vascular cell adhesion molecule 1 (VCAM1)–VLA4 and intercellular adhesion molecule 1 (ICAM1)–LFA1 ligand pairs[6, 17, 18]. This process is orchestrated by chemokines, which provide directional cues for the stem cells to hone to the bone marrow where they settle within a safe haven of stromal cells. Chemokine-mediated mobilization of stem cells reverses the homing extra cellular matrix and stromal cells [5]. Shear stress plays may play a crucial role in altering the adhesion and chemokine profile of motile hematopoietic cells, thereby guiding their homing or mobilization [23-26]. With the ability to provide a 3-dimensional culture condition, and to maintain cells in a constant state of free-fall, the objective is to culture cells in suspension in a low shear stress environment. Studies can depict the changes in cellular function according to cytoskeletal organization and the proteins that link and interconnect filaments to each other, to the cell membrane and other cellular components. Future studies would also lean towards understanding how cytoskeletal cellular disruption could lead to cellular dysfunction, growth arrest, and or necrosis/apoptosis. Based on the scheme that cell cycle machinery is non-hierarchical in nature, this approach may be able to delineate angiogenic processes involved in promoting hematopoietic trafficking, and devise some definition to the chaotic transcriptional networking within the cell.

The goal of this study was to analyze the dynamical properties of mechanical loading via simulated microgravity, its effect on proliferation and differentiation of hematopoietic stem cell (HSPC) growth and its implications in the area of tissue regeneration.

## METHODS

### Collection and Fractionation of Human CD34+ Stem Cells

Cell Cultures HL-60, Jurkat, and KG1-A leukemic cells were cultured in Iscove’s Modified Dulbecco’s Medium (IMDM) supplemented with 10% fetal calf serum (FBS).

**Figure 1:**
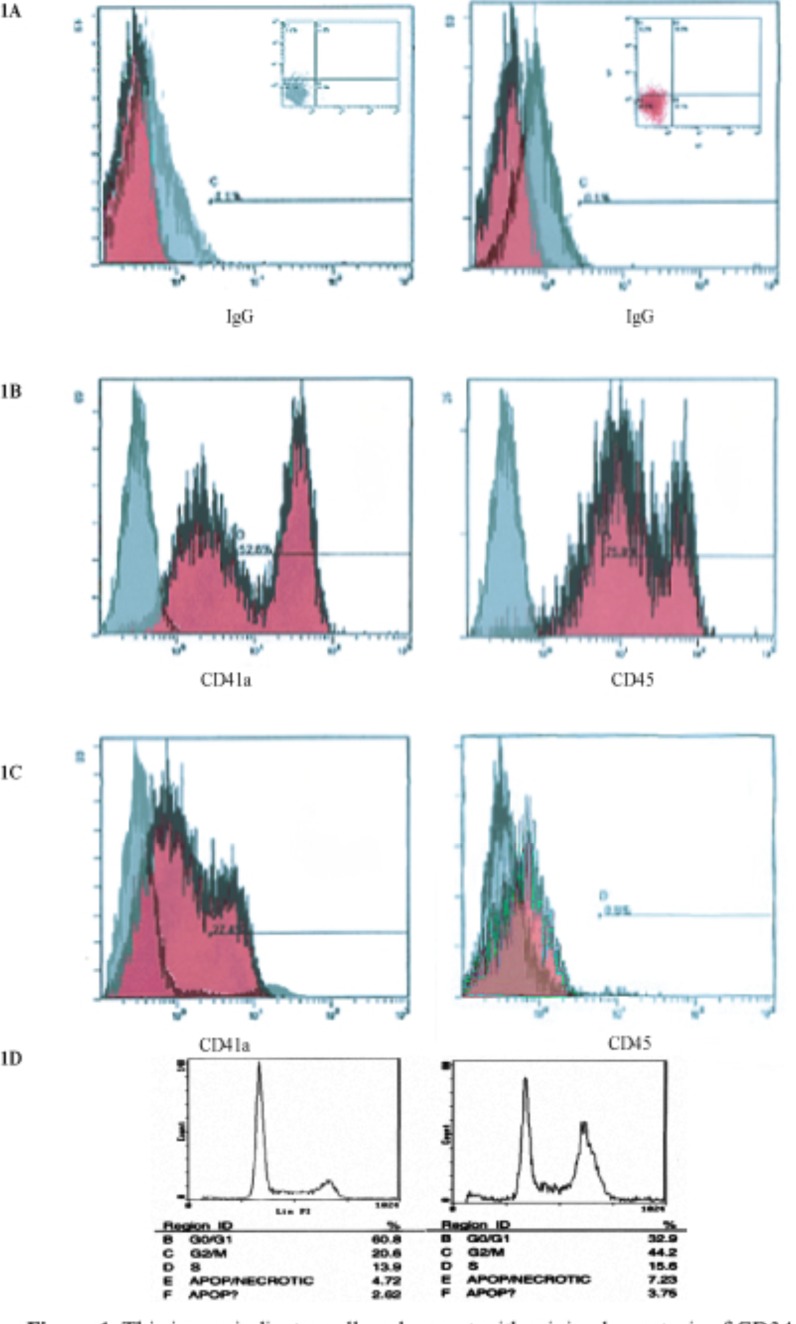
This image indicates cell cycle arrest with minimal apoptosis of CD34+ stem cell in microgravity vs. static 2-D culture conditions Cell cycle analysis shows arrest of 44.2% in G2/M in microgravity displaying the capacity that microgravity has on controlling cell cycle progression and self-renewal capacity. (A) IgG isotype gated control for cells in experiment conditions. Populations of cells are gated in image in top left, also indicated in lower left quadrant of images on top right. (B & C) CD34 PE conjugated transmembrane marker vs. FITC conjugated CD45. Experimental control of static donations, after 72hrs shows cell populations differentiation towards CD45 expression. (D) FACS analysis, 72h experimental conditions.

Bone marrow samples were obtained from normal adult volunteers after receiving informed consent. CD34+ cells were purified from samples using the Miltenyi MinMacs system (Miltenyi Biotech, Auburn, CA), according to the manufacturer’s instructions. Briefly, mononuclear cells were separated using a Ficoll-Plaque (Amersham Pharmacies Biotech, Poscataway, NJ) gradient and incubated with a hapten-conjugated anti-CD34+ monoclonal antibody in the presence of an Fc receptor-blocking reagent. These cells were subsequently incubated with MACS micro beads conjugated to an antihapten antibody and purified using MS+ separation columns (Miltenyi Biotech). Purity of selected CD34 fraction was assessed by flowcytometric analysis using a phycoerythrin (PE)-conjugated monoclonal antibody recognizing different CD34+ epitope. In vitro culture system was then executed and maintained without variation for each specific experimental design. Cell concentration varied depending on bone marrow sample and efficiency of separation, with usual cell counts fluctuating approximately from 1-2 million. CD34+ cells were sustained placed in serum free X-vivo, in a final volume of 2cc, and cultured in Costar Low cluster tissue treated six-well dishes. Cells were then fed accordingly with the use of cytokines, such as Kit Ligand (SCF) and FLK-2, proliferative, and anti-differentiation factors. Concentration of diluted cytokine was modified pertinent to initial cell volume. Cells were cultured for 4 days at 37C before they were exposed to experimental conditions in order to assure purity of cell sample. Flow cytometric analysis was performed at the starting point of each experimental condition in order to assure that the purity of CD34+ cell population is high. Any variation in regards to homogeneity of population was seen through using transmembrane differentiation markers used in analysis represented by figure 1.

### Simulated Microgravity and 1-g Static Cell Culture Conditions

The rotating wall vessel (RWV) was used as a cell culture vessel designed to provide a low-stress cell culture system. Briefly, the RWV system consists of a rotator base, power supply, and a culturing vessel (Synthecon Inc., Houston, TX). Simulated microgravity conditions were created using a reusable 10 ml slow-turning lateral vessel (HARV). An air pump drawed air through a 0.22 μm filter and discharged it into the vessel. Residual air was removed through a syringe port. The HARV was seeded with 2×10^4 cells/ml in x-vivo, supplemented with optimal concentrations of growth factors, IL-3, IL-6, TPO, EPO and SCF in addition to uridine, and rotated at a speed of 8 rpm. In order to evaluate cell growth, the rotating HARV was stopped and the cells were immediately collected by aspiration, centrifuged at 1200 rpm for 5 min, and cell viability was determined by tryphan blue exclusion after a 72-h incubation. Cells were then collected and analyzed for the presence of apoptotic cells and cell cycle analysis by flow cytometry (Coulter Elite Flow Cytometer, Beckman Coulter, Inc., Fullerton, CA) propidium iodide (PI) Apoptosis Kit (Becton Dickinson, Palo Alto, CA), following the manufacturer’s instructions.

### Immunofluorescent Staining

Cells were fixed in 3.7% (v/v) formaldehyde/phosphate-buffered saline (PBS) and permeabilized with 90% methanol/PBS (v/v). Then, cells were incubated with 1 μg/ml of primary monoclonal antibodies (mAb) against VEGFR-1 VEGFR-2 VEGFR-3 (clone 1121, ImClone Systems, Inc., NY), then washed and incubated with secondary FITC-conjugated antibod- ies (1/1000, Vector Laboratories, Burlingame, CA). The samples were mounted in Vectashield containing 4′,6- Diamidino-2-Phenylindole (DAPI) to visualize the nuclei and analyzed by fluorescence microscopy at 400× magnification (Olympus, NJ).

### Western Blot Analysis/ VEGFR’s

Cells were lysed in precipitation buffer (50 mM Tris pH 7.5, 150 mM NaCl, 1% nonidet P-40, 0.1% sodium dodecyl sulfate, and 0.5% deoxycholate). In- soluble debris was removed, and the protein concentration of the supernatant was determined by BCA protein assay kit (Pierce Biotechnology). Cell lysates (100 μg) were separated on 15% SDS–PAGE gels. The protein samples were then transferred to nitrocellulose membrane. Protein expression was confirmed by immunoblotting with anti- bodies raised against VEGFR-1 VEGFR-2, and VEFGR-3 and β-actin (Sigma, St. Louis, MO). After incubation with the appropriate primary antibodies and secondary antibodies.

### Flow Cytometry

Flow cytometric analysis was performed in order to determine cell phenotype pre and post experimental condition in static control and simulated microgravity. This technique was performed at the starting point of every experiment to assure purity of cell isolates from donor tissue. Cells cultured in simulated microgravity were then stained with FITC CD34 antibody vs PE conjugated CD41, CD45 and CD133. The preliminary results are summarized in figure 1, demonstrating preliminary results for both experimental and m-g conditions.

## RESULTS

**Figure 2:**
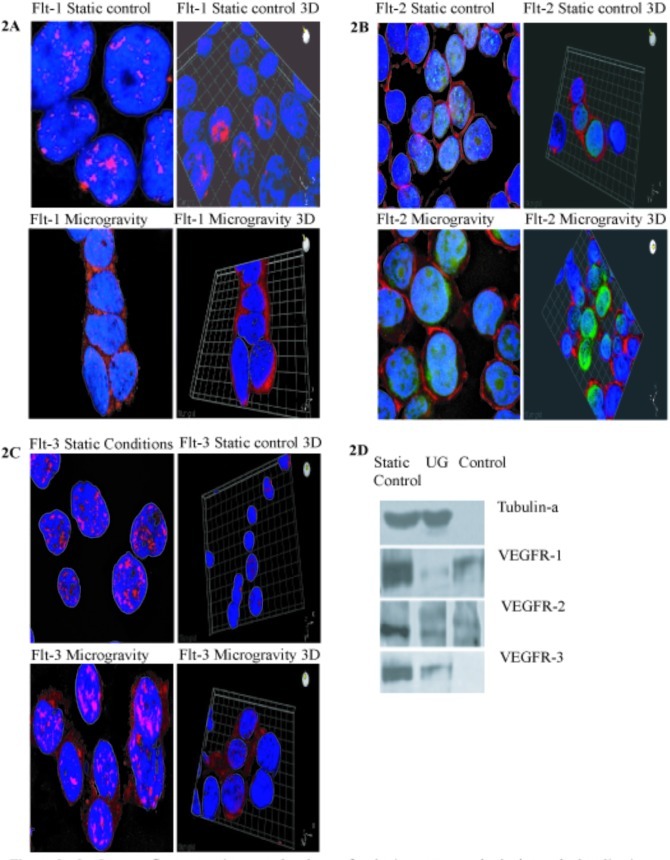
Immunofluorescent images taken by confocal microscopy methods show the localization and the shuttling of VEGFR-1 (Flt-1) VEGFR-2 (Flt-2) VEGFR-3 (Flt-3) in static conditions vs microgravity Protein localization is expressed primarily within the nucleus under normal culture conditions. IN microgravity proteins are shuttled and localized within the plasma membrane. In A and C, PE conjugated antibody for Flt-1 and FLt-3 receptor proteins are designated in red. In B, FITC conjugated antibody Flt-2 protein is indicated in green. (D) Western Blot Analysis of Static Control, Ug and Control samples against Tubulin-a, VEGFR-1, VEGFR-2 and VEGFR-3.

Expansion of and CD34+ in microgravity was assessed after 48-72 hours and compared to static 2-dimensional cultures. Figure 1a specifies IgG isotype-gated control for cells in experimental static conditions. Populations of cells are gated in image in top left, also indicated in lower left quadrant of images on top right. IgG is a standard control used to set the gating for our analysis. IgG vs. transmembrane marker CD34+ which is the population of cells we were testing for. Experimental control of static conditions, after 72hrs culture conditions, (Figure 1b) cell population exhibits differentiation towards CD41a and CD45 expression. There was a major shift in X-Mean from 0.4% to 23.8% strongly exhibiting leukocyte population differentiation. PE conjugated antibody CD34+ vs. CD41a FITC conjugated antiboy showed a major shift of X-mean 0.4% to 18.5%, clearly indicating differentiation towards platelet derived population. Figure 1d specifies cell cycle arrest with minimal apoptosis of CD34+ stem cell in microgravity vs. static 2-D culture conditions. Cell cycle analysis showed arrest of 44.2% in G2/M in microgravity displaying the capacity that microgravity has on controlling cell cycle progression and self-renewal capacity. Figure 1c indicates CD34+PE conjugated antibody vs CD41a and CD45 FITC conjugated antibody. No major shifts occured as indicated by X-mean axis, which stand relatively close, 1.8% to 0.5%. Experimental microgravity conditions after 72hrs incubation time inhibited differentiation. In CD34 PE conjugated antibody vs. CD41a FITC conjugated antibody, microgavity conditions showed no major shifts in peaks retaining CD34 positivity. Immuno-flourescent images taken by confocal microscopy (Figures 2a-2c) showed the localization and the shuttling of VEGFR-1 (Flt-1) VEGFR-2 (Flt-2) VEGFR-3 (Flt-3) in static conditions vs. microgravity. Protein localization was expressed primarily within the nucleus under normal culture conditions. In microgravity proteins were shuttled and localized within the plasma membrane. Western Blot analysis (Figure 2d) showed higher nuclear concentration of VEGFRs as confirmed by immunofluorescence microscopy.

Flow cytometry quantification (figure 3), showed X-mean shifts as indicated in flow cytometry above, quantifying differences in value of CD34+ PE conjugated marker vs. CD45 & CD41a FITC conjugated membrane marker. Static conditions resonated higher values in shift (higher expression of CD45 & CD41a) indicating high differentiation rates as compared to cells in microgravity, which retained their CD34+ expression.

**Figure 3A:**
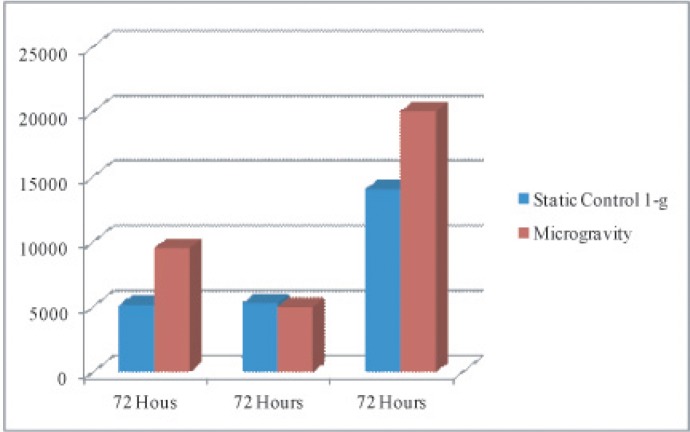
Quantification of expansion rates taken from flow cytometry cell counts (y-axis) in microgravity vs. static condition cultures Cell numbers increase 2-fold in microgravity conditions at 72hrs.

**Figure 3B:**
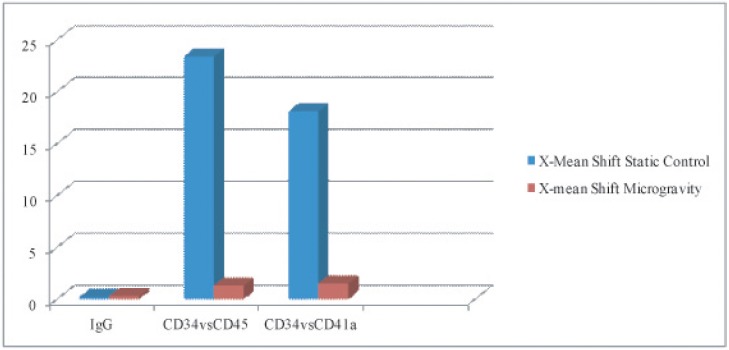
Designates X-mean shifts as shown in flow cytometry above, quantifying differences in value of CD34+ PE conjugated markers vs CD45 & CD41a FITC conjugated membrane marker Static conditions resonate higher values in shift (higher expression of CD45 & CD41a) indicating high differentiation rates as compared to cells in microgravcity, which retain their CD34+ expression.

## DISCUSSION

Studies have portrayed that an autocrine (endothelial-dependent) and a paracrine (endothelial-independent) loop between bone marrow endothelium and cells that reside in the bone marrow, is required in order to sufficiently provide a mode for proliferation and migration. Additionally, literature has eluded to the fact that not only VEGF signals through receptor expression VEGFR-1 (Flt-1) and VEGFR-2 (FlK-2/KDR) specifically regulate endothelial function, but are also involved in hematopoietic stem cell and subsets of leukemia cell development[11, 12] Little is known of the mechanisms by which gravitational forces are converted to biochemical functional responses in cells. The effects of external physiological stresses (blood flow) on hematopoietic stem precursor cells (HSPCs) seem to produce very intricate cellular responses [14, 15]. Our results have identified a novel means to understand the dynamic biomechanical pathways that are involved in the survival of motile and migratory hematopoietic stem precursor cells (HSPC’s). Autocrine as well as paracrine activation loops of VEGF-receptor signaling pathway in HSPC’s have been shown to be essential signaling mechanisms that support cell proliferation and differentiation.

Within the parameters of stem cell plasticity and diversity during development, the hematopoietic system can solely be defined by implication as a dynamic system, providing answers to questions in the laws of occurrence, or nonoccurrence of structural stability. The behavior of dynamical systems contains extreme numbers of coupled elements. Whether genomic, immune, neural, or other evolved systems, the laws governing the behavior of one element are not the same the laws governing the behaviors of other elements, although, thes mechanisms by which these mechanical signals are transduced and converted to biochemical responses are not clearly understood. Our results indicate that VEGR’s specifically VEGFR-1 VEGFR-2 and VEGFR-3 activation occurs in non microgravity conditions. However, functional VEGF-receptors are also found to be expressed not only on cell surface but also predominantly in the nuclei of activated cells. This suggests that mechanical physical and biochemical role of VEGFR signaling may not be solely arbitrated by the conventional model of membrane-bound ligand–receptor interaction. The intracellular trafficking and nuclear localization of VEGF receptors in HSPC’s under static conditions suggest that VEGF receptor intracrine signaling is a potent mediator of differentiative factors. Once inside the nucleus, the potential activity of VEGFR acts a recruiting factor as well a transcription factor itself. These findings set up the stage for enhancing revascularization by exploiting CXCR4, and using simulated microgravity as an agonists and antagonists, respectively. Our studies indicate that living cells are literally hard-wired so that they can filter the same set of chemical inputs to produce different functional out- puts and this mechanism is largely controlled mechanically, through physical distortion of transmembrane adhesion receptors on the cell surface that preferentially transmit stresses to the internal CSK. The reduction in cell proliferation after simulated microgravity was characterized by a slower rate of exit from G2/M phase and by a decrease of the percentage of cells in G/1 and S phases of cell cycle as compared with those in static conditions. Our data shows how cooperation and competition between extrinsic micro environmental chemical signaling (biochemical) and physiological stresses (biomechanical) may be the new frontier in delineating processes in stem cell differentiation and improving techniques in tissue engineering as well as provide a new a model for the development of innovative culture techniques for cell transplant based therapies.

Further studies will not only include elucidating the patterns in receptor trafficking within the cell as a result of simulated microgravity, but as well as the expression of the ligands to these receptors which include; VEGF-A, VEGF-B, VEGF-C, and VEGF-D. Our hematopoietic precursor cells studies also elicit maintenance of stemness. CD41a antigen (gpIIb/IIIa complex) is expressed in platelets and platelet precursors; it acts as a receptor for fibrinogen, von Willebrand factor (vWf), fibronectin, and vitronectin, and it mediates platelet adhesion and aggregation. Our initial data shows that static experimental control displays a major shift toward platelet precursor differentiation, in contrast to m-g conditions. CD45 transmembrane marker staining was also performed, and initial studies showed that population of cells also differentiate towards CD45 expression. Concisely, CD45 antigen is present on all human leukocytes, including lymphocytes, monocytes, granulocytes, eosinophils, and basophils in peripheral blood, and has a major role in the signal transduction, modifying signals from other surface molecules. Further flow cytometric analysis needs to be performed in order to provide additional evidence to verify that our populations of cells in simulated microgravity retain the ability to differentiate. The expression patterns of these proteins and their ligands will be fundamental in understanding its effects on cell cycle activation, proliferation and differentiation, as it applies to transplant based therapy via paracrine/autocrine stimulation. These mechanisms are vital to understanding the cooperative relationship between cells and their surrounding microenvironment. Understanding the expression of these angiogenic triggers will prove to be the cornerstone of bioengineering and the mechanistic machinery that induces tissue regeneration.
